# Towards an Objective Measurement Tool for ADHD-like Traits in Family Dogs: A Comprehensive Test Battery

**DOI:** 10.3390/ani14131841

**Published:** 2024-06-21

**Authors:** Barbara Csibra, Vivien Reicher, Melitta Csepregi, Kíra Kristóf, Márta Gácsi

**Affiliations:** 1 Department of Ethology, Institute of Biology, Eötvös Loránd University, Pázmány Péter Sétány 1/C, 1117 Budapest, Hungary; 2 Doctoral School of Biology, Institute of Biology, Eötvös Loránd University, Pázmány Péter Sétány 1/C, 1117 Budapest, Hungary; 3 Clinical and Developmental Neuropsychology Research Group, Research Centre for Natural Sciences, Institute of Cognitive Neuroscience and Psychology, Magyar Tudósok Körútja 2, 1117 Budapest, Hungary; 4 HUN-REN—ELTE Comparative Ethology Research Group, Pázmány Péter Sétány 1/C, 1117 Budapest, Hungary

**Keywords:** ADHD, behavioural test, inattention, hyperactivity, impulsivity, questionnaire, animal model, domestic dog

## Abstract

**Simple Summary:**

Attention-deficit/hyperactivity disorder (ADHD) is a prevalent neurodevelopmental disorder in humans, characterised by inattention, hyperactivity, and impulsivity. Evidence suggests that family dogs can exhibit behaviours similar to symptoms of ADHD seen in humans. While past research mainly used questionnaires to assess ADHD-like behaviours in dogs, we aimed to create a simple behavioural test battery covering key ADHD symptom dimensions: inattention, hyperactivity, and impulsivity. Using our previously validated questionnaire (Dog ADHD and Functionality Rating Scale–DAFRS; 2024) in a study involving 59 dogs, we correlated owner-rated DAFRS scores with behavioural test outcomes to further validate our questionnaire. In a subsample of 38 dogs, we also determined whether there is a difference in how owner- and trainer-rated ADHD scores are linked with the test variables. Our test battery included attention, plush dog, leash, and sit tests, all showing significant correlations with the relevant questionnaire scores in an expected direction. Both owner- and trainer-rated ADHD questionnaire factor scores showed correspondence with the relevant behavioural test variables. Dogs’ training status only showed association with the sit test. The test battery proved to be consistent in time; test–retest analyses demonstrated moderate-to-excellent agreement across all tests. Our findings endorse the validity of our questionnaire and behavioural tests, providing valuable tools for assessing inattention, hyperactivity, and impulsivity in dogs.

**Abstract:**

Family dogs exhibit neuropsychological deficits similar to attention-deficit/hyperactivity disorder (ADHD) symptoms in humans. Questionnaire methods have mostly been used to assess ADHD-like behaviours in dogs. In addition to our validated questionnaire (Dog ADHD and Functionality Rating Scale–DAFRS; 2024), we developed a simple behavioural test battery covering the ADHD symptom domains (i.e., inattention, hyperactivity, and impulsivity) in dogs. Our main aim was (i) to provide a final external validation step to the DAFRS by examining its associations with the test variables (*N* = 59); and (ii) to compare owner- and trainer-rated factor scores’ associations with the test variables (*n* = 38). We developed four tests covering the ADHD symptom domains: the attention test (inattention), the plush dog test (impulsivity), the leash test, and the sit test (hyperactivity). All four behavioural variables correlated with their respective questionnaire scores, i.e., the strongest for hyperactivity, and the least strong for inattention. Both owner- and trainer-rated scores (*n* = 38) correlated with the relevant test variables in an expected direction. Dogs’ training status was linked only to the sit test results. Test–retest analyses (*n* = 34) indicated moderate-to-excellent agreement across all behavioural variables. Our findings support the validity of our novel human-analogue questionnaire for dogs as the behavioural tests strongly correlate with the relevant questionnaire scores, indicating that the two constructs together can effectively assess inattention, hyperactivity, and impulsivity in dogs.

## 1. Introduction

In humans, attention-deficit/hyperactivity disorder (ADHD) is a neurodevelopmental disorder characterised by persistent patterns of inattention, hyperactivity, and impulsivity that significantly impair functioning in various life domains [1,2]. Assessing ADHD symptoms in humans involves a multi-method approach using questionnaires, clinical interviews, and behavioural assessments [3]. Parent- and/or teacher-reported behaviour rating scales are the primarily used tools to measure the behavioural criteria for ADHD and the presence of related impairments in different settings, as children are unable to accurately assess their own symptoms and their severity [3,4,5,6]. In theory, the DSM-V criterion strictly outlines which symptoms must be met to establish the diagnostic criteria [1,2]; however, in practice, it relies heavily on subjective reports and opinions rather than objective measurements [7]. The sole reliance on parents as informants on symptoms raises concerns about the reliability of the diagnostic criteria and their subjective nature [8,9]. This indicates that diagnosing ADHD in humans is a complex, multi-method process, and itshould contain an objective assessment of behaviour, e.g., with behavioural tests, –as symptoms are mainly manifested in behaviour–, even if questionnaires are the primary tools used to assess symptoms. Since there is no universal, specific behavioural test for ADHD, one approach to externally validate parent and teacher reports involves the direct observation of a child’s behaviour in key settings pertinent to the diagnosis, including test sessions and school classrooms [10]. During test sessions, evaluators can directly observe the behavioural manifestations of ADHD symptoms (as described in the DSM), while children engage in structured cognitive or academic tasks, with the application of coding systems and operational definitions to quantify targeted behaviours [1,2,10]. In addition, neuropsychological tests are applied to assess different aspects of executive functions related to ADHD [11,12,13] such as impairments related to sustained and selective attention, cognitive flexibility, working memory, and inhibition [13,14].

A growing body of research suggests that family dogs may serve as a potential animal model of ADHD as, similarly to humans, dogs naturally exhibit phenotypic variability with regard to attention, activity, and impulsivity [15,16,17,18,19]. In veterinary practice, an increasing number of cases report dogs exhibiting behaviours similar to human ADHD, often diagnosed and treated as “Hypersensitivity–Hyperactivity syndrome” (HSHA) [20,21,22]. While veterinarians commonly refer to this syndrome as “canine ADHD”, it is doubtful whether HSHA is truly analogous to human ADHD in dogs. The replicability of the results published so far in the field of “canine ADHD” is challenging, given the absence of a uniform definition and diagnostic criteria [22,23].

Currently, questionnaires are mainly used to scientifically assess inattention and hyperactivity/impulsivity in dogs [18,24]. The Dog ADHD Rating Scale (Dog ARS; [18]) is the most extensively used rating scale for evaluating natural variability in inattention and hyperactivity/impulsivity in dogs. Yet it does not include a functionality assessment, which is an essential component in human ADHD assessments [25]. In our prior research, we developed the Dog ADHD and Functionality Rating Scale (DAFRS), which measures inattention, hyperactivity, and impulsivity separately, including items on functional impairments [19]. The DAFRS has demonstrated psychometric validity and reliability; however, it is important to note that external validation through behavioural tests has not been previously conducted. This underscores the need for additional research to explore and validate the DAFRS in behavioural contexts, contributing to a more comprehensive understanding of its applicability and effectiveness in assessing inattention, hyperactivity, impulsivity, and functionality in dogs.

Regardless of some attempts to assess ADHD-like behaviour in dogs using behavioural tests [20,26,27,28], there is currently no objective behavioural test that specifically measures and covers all the ADHD symptom dimensions in dogs. Notably, Kubinyi and colleagues developed a simple test battery: the activity–impulsivity behavioural scale (AIBS) [27]. The test measures four tests: “spontaneous activity” on leash (owner holding the dog, which can move freely on the leash, for one minute); “separation and play” (separation from the owner; the experimenter initiates play for half minute); “lying on the side” (owner tries to turn the dog on its side and keep the dog in this position for half minute); and “approaching the owner” (owner hides; after half minute, the experimenter releases the dog) [27]. The name of the behavioural test battery can be misleading, as these tests, aside from the “spontaneous activity” test, may not necessarily uniquely measure inattention or hyperactivity/impulsivity; for instance, the “lying on the side” test could significantly depend on the dog’s training level, while the “separation and play” and “approaching the owner” tests might be influenced by the dog’s relationship with its owner. Nevertheless, this remains the only behavioural test battery that has attempted to evaluate inattention and hyperactivity/impulsivity through behavioural measures. The study revealed a relationship between the Dog ARS scales and the AIBS score, although it is important to note that the scores from the tests were summed and added together, so the total score for the test battery was correlated with each questionnaire factors, rather than specifically for each test (e.g., spontaneous activity–hyperactivity/impulsivity) [27].

A recent study developed an objective video-based assessment of ADHD-like dog behaviour, applying machine learning [20,26]. A machine learning classifier was trained to distinguish between dogs clinically treated in the context of ADHD-like behaviour and a typical control group, and it showed 81% accuracy [20,26]. However, the precise diagnostic criteria used to differentiate between groups was not provided. While the method holds promise, its technical implementation might necessitate equipment and expertise, raising questions about its broader practical applicability.

It should be noted that behavioural measurements of ADHD-like traits in dogs within the veterinary practice may be burdened by the fact that dogs might experience elevated stress levels in the clinic or in the veterinary room, if they have associated negative experience with prior veterinary visits. For instance, previous studies on dogs’ heart rates have shown that the location of the experiment—and, potentially, the stress level associated with the location—can affect the results. In a veterinary office, the average heart rate was 124 beats/min [29], while during a behavioural experiment with minimal physical activity and stress, it was 69–95 beats/min [30,31,32]. Similarly, stress and frustration can make dogs hyperactive, impulsive, and inattentive [17], which might lead to inaccurate measurements of these traits. These factors are often neglected in behavioural experiments but may influence the diagnostic measurements for ADHD in dogs (and the results described so far within the field). Thus, there is a need for behavioural assessments that remain as much as possible uninfluenced by environmental factors.

From the three traits, impulsivity has been the most extensively studied in dogs [33,34,35,36,37,38,39,40]. Despite the psychological concept of impulsivity (behaviour without adequate forethought, predisposition towards sudden reactions to external stimuli without regard for the possible negative consequences [41]), in previous studies, the focus has primarily been on inhibitory control linked to impulsivity (such as reduced ability to tolerate delays [34,35] or behavioural disinhibition in a touch-screen task [39,40]), rather than measuring the sudden reaction to external stimuli. It is also likely that impulsivity is difficult to consistently measure in dogs because the applied tests are more likely to capture different aspects of the trait, as different tests measuring impulsivity may not correlate with each other [35] or may present inconsistent results [33,36,37].

Recently, we developed and validated an ADHD questionnaire for measuring inattention, hyperactivity, impulsivity, and functionality in family dogs (Dog ADHD and Functionality Rating Scale: DAFRS; [19]). The rating scale has a stable factor structure and good test–retest reliability, where both owners and trainers can evaluate dogs [19]. Even though recent behavioural studies suggest that dogs may exhibit problem behaviours resembling to ADHD [21,22,23,42], so far, the DAFRS has not been used to distinguish between normal and pathological levels of impulsivity, hyperactivity, and inattention [19].

While questionnaires can serve as valid and effective tools, their reliance on subjective responses poses a challenge. Therefore, there is a high demand for behavioural tests which can be used to objectively assess the behavioural manifestations of ADHD characteristics and to screen for extreme individuals. Such behaviour tests could also serve as an external validation of the DAFRS questionnaire. As highlighted before, currently, there is no behavioural test that comprehensively covers all domains of ADHD-like behaviour in dogs and is easily implementable and applicable in practice. In this case, simpler and shorter tests that focus on individual domains of ADHD-like behaviours (such as inattention, hyperactivity, impulsivity) can be advantageous. Utilising multiple tests may introduce a massive order effect and could potentially mask the dog’s natural behaviour in complex, lengthier assessments, making these less practical to apply [43].

### Current Study

Our aim was (i) to add a final validation step to the DAFRS questionnaire by developing a behavioural test battery measuring inattention, hyperactivity, and impulsivity; (ii) to investigate the correlations between the results of the behavioural tests and the owner- vs. trainer-rated DAFRS questionnaire factor scores (i.e., for inattention, hyperactivity, and impulsivity), also accounting for training status.

Specifically, we formulated the following questions:

**1. Do the data from the questionnaire scores and the relevant behavioural test variables correlate in terms of inattention, hyperactivity, and impulsivity, accounting for training status? (*N* = 59)**

Based on earlier findings in dogs and humans [3,18,27], we expected that both tools could capture inattention, hyperactivity, and impulsivity in dogs; thus, the two measurements would correlate. We expected that the behavioural tests assessing the specific ADHD domain would correlate with the relevant questionnaire scores (e.g., activity test with hyperactivity score). As dogs’ training status may affect the results of the different tests, we included this variable in our analyses.

**2. Do the owner and trainer evaluations show similar associations with the behavioural tests? (*n* = 38)**

Based on our prior research, both the owner and the trainer should be able to provide an accurate depiction of the dog’s behaviour; therefore, we can anticipate quite similar correlation coefficients when comparing raters [19]. However, since for both parent–teacher and owner–trainer comparisons revealed fair-to-moderate agreements [25,44,45], when comparing owner–trainer questionnaire evaluations, it is conceivable that the trainers evaluate much more objectively, resulting in a stronger correlation between their evaluation and the behaviour test, similarly to findings in humans [46,47]. Concerning inattention, parent–teacher and owner–trainer questionnaire evaluations are typically less strong in terms of agreement [45]. Thus, it is also possible that for specific dimensions, one evaluator’s rating will be more strongly correlated to the relevant behavioural test than the other, and vice versa.

**3. Are the behavioural test variables reliable across time (test–retest reliability) regarding the different tests? (*n* = 34)**

Although examining test–retest reliability is often not included in studies of dog behaviour, generally, test–retest reliability results have exhibited considerable variability in behavioural measurements of dog personality [48]; however, there is typically a degree of consistency observed over time, as outlined in previous research [27,48]. We expected that the variables measured in the behavioural tests would be consistent over time, based on prior behavioural test–retest measurements of activity and impulsivity in dogs [27]. However, there may be stronger agreement in measurements of hyperactivity and impulsivity than in measurements of inattention, similarly to our previous questionnaire results [19,25].

## 2. Materials and Methods

### 2.1. Ethics Statement

All experimental protocols were approved by the Hungarian “Scientific Ethics Committee for Animal Experimentation” (PE/EA/00035-4/2023) and were conducted in accordance with Hungarian regulations on animal experimentation and the guidelines for use of animals in research as outlined by the Association for the Study Animal Behaviour (ASAB). Prior to participation, owners and trainers received detailed information about the aims, circumstances, and features of the experiments. Informed consent was obtained from all subjects involved in the study. All questionnaires were carried out in accordance with the relevant guidelines, and regulations and were approved by the United Ethical Review Committee for Research in Psychology (EPKEB; Ref. no.: 2023-04).

### 2.2. Subjects

Participants were 59 family dogs (*M*_age_ = 3.86 years, *SD* = 2.35, range: 1 to 9 years) of 22 different breeds and 22 mongrels (32 male and 27 female; 14 intact and 45 spayed). Subjects were recruited and pre-selected based on their ADHD questionnaire scores on the DAFRS [19]. In addition to trying to cover the full range of ADHD questionnaire scores (range of scale scores: inattention: 0–18 points; hyperactivity: 0–12 points; impulsivity: 0–21 points; ADHD total score: 0–51 points), we have specifically tried to include high-scoring dogs in our tests, particularly those on the extreme high end of the ADHD score spectrum (e.g., had higher than 30 points on the ADHD total score, based on the distribution of the scale points in our earlier study [19]). In the present sample, the range of scale scores were inattention: 0–17 points (*M* = 4.47, *SD* = 3.56); hyperactivity: 0–12 points (*M* = 5.93, *SD* = 3.48); impulsivity: 0–21 points (*M* = 9.63, *SD* = 6.05); and ADHD total score: 0–49 points (*M* = 20.03, *SD* = 11.44). Owners and their dogs were recruited through the Department of Ethology participant pool and website, through popular social networking sites, and via snowball sampling. Dog trainers (*N* = 38) were recruited via the dogs’ owners (owners were asked to involve the trainer working with their dog).

To obtain a measurement of test–retest reliability, the dogs which were still available were retested 5.1–14.5 months (average: 9.9 months) after the first test session (*n* = 34). This subsample (*M*_age_ = 3.85 years, *SD* = 2.52; range: 1 to 8 years) consisted of 13 different breeds and 15 mongrels (18 male and 16 female; 8 intact and 26 spayed dogs).

### 2.3. Measures

#### 2.3.1. Rating Scale Measures

Dogs’ owners and dog trainers were asked to complete questionnaires online that consisted of the following rating scales and questions.

***Inattention, Hyperactivity, and Impulsivity:*** Individual differences in inattention, hyperactivity, and impulsivity were measured using the Dog ADHD and Functionality Rating Scale (DAFRS; [19]), both the owner version and the trainer version (for dogs who had trainer ratings), which is a 17-item (6 items measuring inattention, 4 items measuring hyperactivity, and 7 items measuring impulsivity) owner- and trainer-reported measure of dogs’ level of attention, hyperactivity and impulsivity. Apart from the three main ADHD dimensions, the rating scale consists of questions pertaining to the dogs’ functioning in connection with inattention, hyperactivity, impulsivity, and aggression. Owners indicate the frequency with which their dog behaves as described in each item (ranging from “never” = 0 to “very often” = 3). Examination of the measurement’s psychometric properties (with over 1100 dogs representing over 100 different dog breeds) indicated evidence for its internal consistency and validity.

***Training status:*** We included owner-reported training status in our analysis, as we hypothesised that training might have an effect on dogs’ behaviour in the tests, and it was also shown to be associated with differences in canine inattention, hyperactivity, and impulsivity [18,19,25]. Differences in training status were indexed as “basic” (basic obedience training), “intermediate” (higher level obedience training), or “advanced” (advanced obedience training) training. In our sample, 27 dogs had basic, 20 had intermediate, and 12 had advanced training.

#### 2.3.2. Behavioural Tests

We formed a series of behavioural tests to cover inattention, hyperactivity, and impulsivity traits in dogs. Owners were asked not to participate in tiring activities with the dog (e.g., playing, dog school, training, sporting, running) before the behavioural experiment. Before the tests, the experimenter explained the aim of the behavioural tests and gave instructions for the dog owner for each test. All experimental procedures took place at the Eötvös Loránd University, Department of Ethology, in a 3 m × 6 m experimental room. The tests were performed in a fixed order, as presented below, to ensure consistency and reduce the complexity of the experimental design, aligning with our aim to develop a simple behavioural test battery. Given that certain tests were expected to introduce stress and/or fatigue, we used a fixed order to control for these effects consistently across all subjects. Additionally, fixed-order tests facilitate easier replication in subsequent studies as researchers can follow the same sequence, leading to better comparability across different studies.

**Figure 1:**
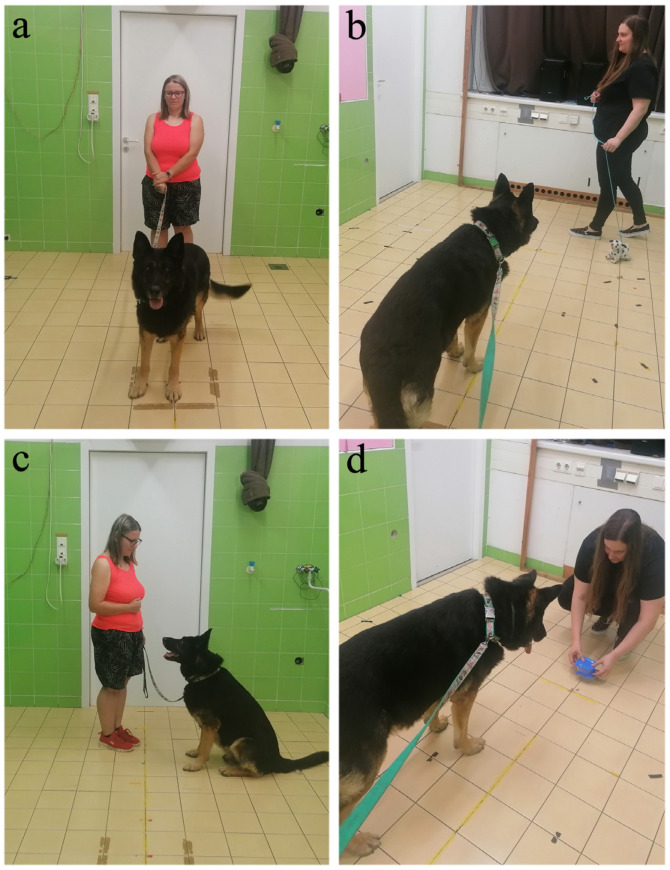
Depiction of the experimental setup of the behavioural test: (**a**) leash test; (**b**) plush dog test (first part); (**c**) sit test; (**d**) attention test.

**1. *Leash test:*** The present test was adopted based on earlier research by Kubinyi et al. [27]; aimed to measure dogs’ activity level (see Figure 1a). In prior research, the behavioural test correlated with the Dog ARS subscales [18], both with inattention and activity–impulsivity [27], and this test seemed to be simple to perform without any pre-training.

The owner (O) was asked to stand still, without paying special attention to the dog, while holding the dog on a 1.5 m leash. The dog was allowed to move freely within the range of the stretched leash and was not corrected or rewarded for any behaviour. To prevent leash correction by the O, the experimenter (E) asked the O to hold the wrist of the hand that holds the leash. The test lasted for 2 min. E stepped back and stayed at a distance of 3 m from the dog, measuring the 2 min with a stopwatch.

**2. *Plush dog test:*** This test aimed to measure dogs’ impulsivity. To elicit an impulsive response, we used a small plush dog (25 × 25 cm) on a leash as a strange, external stimulus.

When the second minute of the previous test passed, E asked O to remain still and not to talk to the dog (e.g., not ask the dog to sit), only holding the leash while the dog could go freely. E took a plush dog on a leash from outside of the room and started to walk it in a straight line, 3 m away from the dog, measuring 1 min with a stopwatch. The dog was allowed to move freely within the range of its leash and was not corrected or rewarded for any behaviour, until the end of the first minute of the test (see Figure 1b).

After 1 min passed, E stopped, put the plush dog in front of her, remaining still, and stayed there for 1 min. This time, after E stopped moving with the plush dog, O was allowed and instructed to control the dog, but only by saying the dog’s name a maximum two times if the dog wanted to interact (lunging, barking, pulling the leash, growling) with the plush dog. We aimed to measure whether the dog was able to stop the reaction when the owner asked for it. After 1 min passed, E took the plush dog outside.

***Exploration:*** The purpose of this phase was to ensure that the dog was aware that the plush dog had been removed from the room after the previous test and to allow the dog to calm down during the exploration period if it had shown a reaction in the plush dog test. E asked O to release the dog from the leash, allowing the dog to explore the lab for 2 min. After 2 min passed, E asked O to put the leash back on the dog. This phase was not considered as a test in our analysis.

**3. *Sit test:*** This test aimed to measure hyperactivity by measuring whether the dog could remain still (see Figure 1c).

O was asked to command (verbally and/or with hand gestures) the dog to sit down and then to stay in front of the dog, quietly, while holding the dog on a leash (1.5 m). O was not allowed to use commands referring to “Stay” or to show the hand signal afterwards. If the dog refused to sit down or got up, changed, or left the position during the test, O had to command the dog again to sit down. The only exception was if the dog laid down from the first sitting position, in which case, O did not have to command the dog to sit up again (we were more interested in how well the dog could remain still). O was instructed by E to use as few commands as they could during the test to move the dog into the sit position. O was not allowed to physically handle the dog or correct it with the leash to help it to move into the sitting position. Test was terminated after 1 min; then, O was asked to leave the room with the dog until E set up the lab for the next experiment.

**Figure 2:**
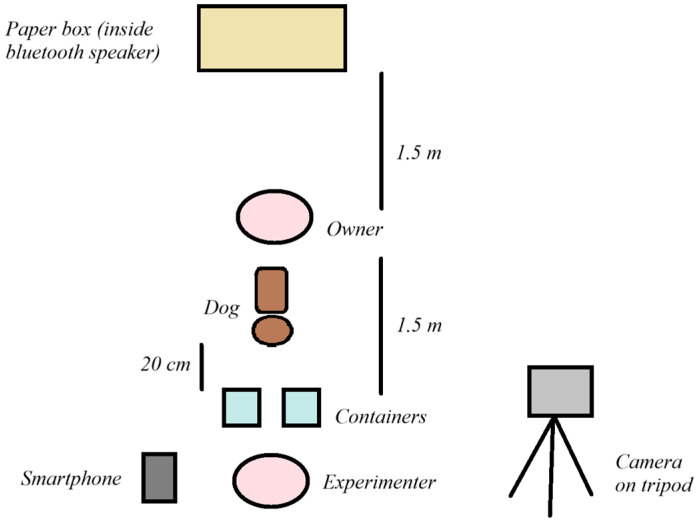
Depiction of experimental setup of the attention test showing the location of the experimenter, the owner, the dog, and the experimental tools used in the test.

**4. *Attention test:*** This test aimed to the measure dog’s attention. Human behavioural tests measuring inattention related to ADHD often use a distractor sound as an external stimulus [49]. In a similar manner, we developed an attention test for dogs in which we introduced a distractor sound during the test. The experimental setup is shown in Figure 1d and Figure 2.

A tripod with a camera was set up before the test in the room. A Bluetooth speaker was hidden in a 20 × 30 × 20 cm paper box in the room before the test (behind the dog and O), which was controlled via a smartphone by E. O was asked to stand in the middle of the room with the dog on a marked spot (1.5 m distance from E, who was in front of the dog, and 1.5 m distance from the sound source, which was in a paper box, behind the dog and O). O was asked to stand motionless in the same place without looking at the dog or talking to the dog, while holding the dog on leash (1.5 m). E placed a piece of dog food in a transparent plastic container and offered it to the dog (while holding the container in her hands). The test was terminated if the dog did not accept the food. The food was offered to induce the dog to be interested in the containers (which were manipulated later during this test) and to catch its attention. After this, E did not give any food for the dog; the two containers were empty during the test as we wanted to minimise the possible influence of the dogs’ motivation for food in this test. After E offered the food to the dog, and before the start of the test, E put down the smartphone next to her and kneeled on the floor in front of the dog (20 cm between the dog and the containers). E started a 1-min-long sound playback, which started with 20 s of silence, was followed by 20 s of distractor sound, and, lastly, was followed by 20 s of silence. The distractor sound was a recording of the sound of a vacuum cleaner. The recording was a continuous sound; the noise of the vacuum cleaner alternated between fading out and increasing. We selected the vacuum cleaner noise as a distractor because it is likely familiar to all dogs. Additionally, we ensured that the sound was not played too loudly to elicit fearful reactions. The distractor sound is openly accessible via FigShare (see link in Supplementary Materials).

After starting the recording, E put down two empty transparent plastic containers with lids onto the floor, right in front of herself and in front of the dog. E tried to catch the dog’s attention by calling its name and saying “Look!” while initiating eye contact with it. Afterwards, E manipulated and fiddled with the containers for 1 min (changed the lids on the two containers and changed their location, lifting one container up and placing it next to the other, and putting one container on the top of the other). E tried to catch the dog’s attention by using the above-mentioned cues after every 20 s, regardless of whether the dog was paying attention to her or not. The test was terminated after 1 min (when the sound recording was over).

***Retest measures:*** Retests were intended to measure the tests’ consistency over time. The same procedure (leash test, plush dog test, exploration, sit test, and attention test) was followed as described above on a second occasion in the same room with the dogs which were available for retest measurements (see Subjects).

***Behavioural observation and coding procedures:*** To assess differences in dogs’ attention, activity, and impulsivity, the behavioural tests were videotaped and coded for predefined behavioural responses, with a 0.2 s inspection of the recordings using Solomon Coder (© András Péter, http://solomoncoder.com/ (accessed on 29 May 2024)). The behavioural variables of interest and their definitions are presented in Table 1. Inter-rater reliability (Cohen’s Kappa) was calculated for all behavioural variables by double-coding 19 dogs’ recordings out of 59 (32% of the total sample), and it indicated almost perfect agreement between the two raters (overall κ = 0.850; activity κ = 0.864; looking at experimenter κ = 0.763; leaving position κ 0= 0.925).

#### 2.3.3. Data Analysis

SPSS V27.0.1.0 was used for all analyses. As some of the examined variables showed non-normal distribution, we performed Spearman’s rank correlations. As training may have an effect on the behaviour observed, we also included training status in the correlations, and when training showed association with a variable, a partial correlation was conducted in order to disentangle the effect of training status. To control for multiple comparisons, Benjamini–Hochberg correction was conducted, and results based on the adjusted *p*-values are reported.

Regarding the attention test, seven video recordings were not saved due to technical issues, resulting in an adjusted sample size of *n* = 52 for the “looking at experimenter” data in the case of the total sample and *n* = 33 in the case of the subsample of the owner and trainer ratings.

Analyses of test–retest reliability of the behavioural tests were conducted on a subsample size of *n* = 34. Intraclass correlation coefficients (ICC) with corresponding 95% CIs were computed for the test–retest reliability analyses (3, k consistency) on the examined behavioural variables. ICCs can range from −1 to 1 and, in accordance with convention, were interpreted as follows: 0 ≤ 0.3 as poor; 0.3 ≤ 0.5 as fair; 0.5 ≤ 0.7 as moderate; 0.7 ≤ 0.8 as strong; and >0.8 as excellent or almost perfect [50].

## 3. Results

**Figure 3:**
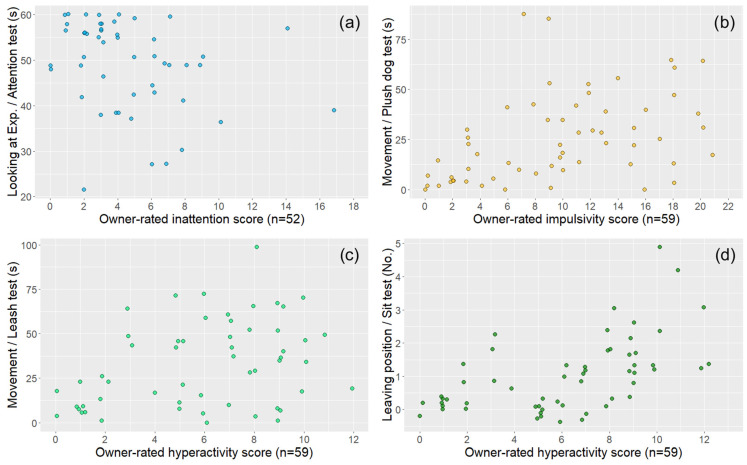
Associations between owner-rated DAFRS questionnaire scores (total sample, *n* = 59) and the behavioural variables of tests used in the present study: (**a**) looking at the experimenter in the attention test—owner-rated inattention score; (**b**) movement in the plush dog test—owner-rated impulsivity score; (**c**) movement in the leash test—owner-rated hyperactivity score; (**d**) leaving position in the sit test—owner-rated hyperactivity score. The coloured circles represent the subjects. All the associations were significant and are presented based on Table 2.

All four behavioural variables showed associations with the relevant owner-reported questionnaire subscale score data (*N* = 59; Table 2 and Figure 3).

**Figure 4:**
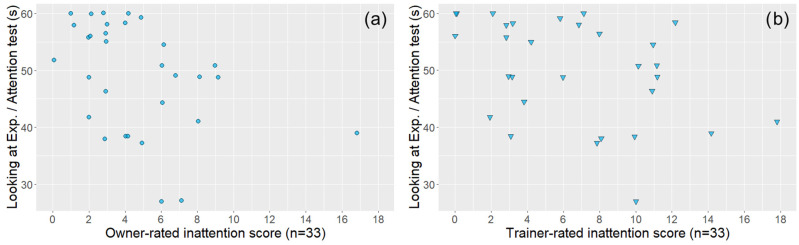
Associations between owner-rated (**a**) and trainer-rated (**b**) DAFRS inattention questionnaire scores (subsample, *n* = 33) and the looking at the experimenter variable of the attention test. The coloured circles (owner ratings) and triangles (trainer ratings) represent subjects. All the associations were significant and are presented based on Table 2.

**Figure 5:**
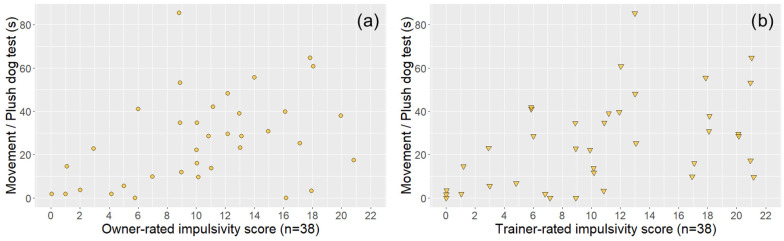
The associations between owner-rated (**a**) and trainer-rated (**b**) DAFRS impulsivity questionnaire scores (subsample, *n* = 38) and the movement variable of the plush dog test. The coloured circles (owner ratings) and triangles (trainer ratings) represent subjects. All the associations were significant and are presented based on Table 2.

**Figure 6:**
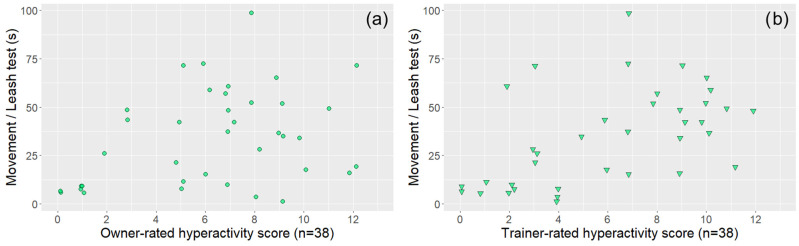
The associations between owner-rated (**a**) and trainer-rated (**b**) DAFRS hyperactivity questionnaire scores (subsample, *n* = 38) and the movement variable of the leash test. The coloured circles (owner ratings) and triangles (trainer ratings) represent subjects. All the associations were significant and are presented based on Table 2.

**Figure 7:**
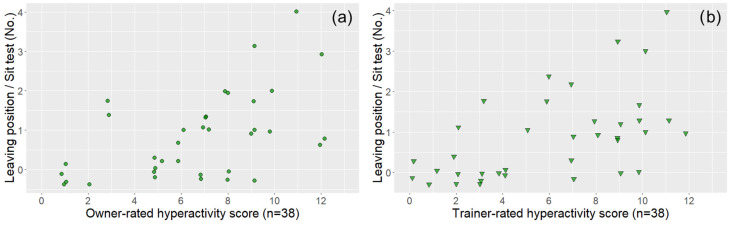
The associations between owner-rated (**a**) and trainer-rated (**b**) DAFRS hyperactivity questionnaire scores (subsample, *n* = 38) and the leaving position variable of the sit test. The coloured circles (owner ratings) and triangles (trainer ratings) represent subjects. All the associations were significant and are presented based on Table 2.

In the case of dogs that also had a trainer evaluation (*n* = 38), both trainer- and owner-rated questionnaire subscale scores were associated with the relevant behavioural variables, although in one case (Hyperactivity score—Movement in the leash test), the significance level was not the same (Table 2 and Figure 4, Figure 5, Figure 6 and Figure 7).

The training level correlated only with one behavioural variable (leaving position in the sit test) in the case of both the total sample (*N* = 59; *r* = −0.344, *p* = 0.008) and the subsample (*n* = 38; *r* = −0.472, *p* = 0.003). After conducting partial correlation analysis by training, significant associations with the leaving position variable persisted in all three cases; with owner-rated hyperactivity score (*N* = 59; *r* = 0.582, *p* < 0.001), with trainer-rated hyperactivity (*n* = 38; *r* = 0.539, *p* = 0.001) and the corresponding owner-rated hyperactivity score (*n* = 38; *r* = 0.530, *p* = 0.001).

Regarding the test–retest (*n* = 34) results, ICCs represented moderate to almost-perfect consistency between the two test occasions: looking at the experimenter in the attention test showed moderate consistency (ICC = 0.577; 95% CIs = [0.153; 0.789]); movement in the leash test showed excellent consistency (ICC = 0.801; 95% CIs = [0.601; 0.900]); movement in the plush dog test showed almost-perfect consistency (ICC = 0.837; 95% CIs = [0.673; 0.918]); and leaving position in the sit test showed strong consistency (ICC = 0.752; 95% CIs = [0.504; 0.876]).

## 4. Discussion

The primary objective in our research was to strike a balanced approach in meeting two specific requirements; (i) to add a final external validation step to our recently developed questionnaire, the DAFRS [19]; and (ii) to develop a behavioural test battery of the utmost simplicity to comprehensively and objectively assess inattention, hyperactivity, and impulsivity. Our findings indicate that we have successfully balanced between these challenges.

All behavioural test variables showed significant correlations with the relevant owner-reported questionnaire subscale scores in the expected directions (e.g., a higher inattention score associated with less looking at the experimenter in the attention test), indicating that both constructs can capture inattention, hyperactivity, and impulsivity in dogs. Given that there was a single test session, and each test was characterised by a single variable, as well as the potential influence of confounding parameters, we did not anticipate exceptionally strong correlations with the questionnaire scales. This expectation is supported by studies on ADHD in humans, which reported moderate correlation coefficients ranging from 0.2 to 0.5 for questionnaire–behavioural test associations [51,52]. Similarly, for dog personality traits [48], inattention, and activity–impulsivity [27], the correlations ranged from 0.2 to 0.6. Dog owners’ biases or experiences may have influenced their questionnaire responses, potentially overestimating/underestimating the dog’s inattention, activity, and impulsivity; whereas the behavioural tests can measure these traits more objectively. At the same time, behavioural tests may only reflect a momentary state; thus, limited generalisations can be made based on their results. This poses challenges as the lower correlations between the questionnaire and the behaviour test (e.g., in the case of inattention) may be attributed to the drawbacks of either or both methods. Based on our findings, it is not possible to disentangle the two tools’ specificity and sensitivity. Further research is needed to clarify the advantages and disadvantages of the behavioural test battery and the DAFRS questionnaire in accurately measuring ADHD trait dimensions. Thus, further investigation is required to assess the complementarity and overlap of these two instruments, which could further enhance their validity.

The measured behavioural variables showed correlations with both owner and trainer ratings; however, trainers did not consistently evaluate dogs’ behaviour more accurately across all four tests. While significant correlations were found with both owner and trainer ratings, a higher correlation coefficient showed up for trainer ratings regarding the association between the hyperactivity score and the movement in the leash test. Based on Figure 6, the distribution of the trainers’ scores better fit the observed behaviour than owners’ scores, which suggests that dog trainers can better assess dogs’ hyperactivity. In comparing activity levels and temperament, trainers may have an advantage as they understand which breeds are naturally more active/energetic and can distinguish between typical and excessive activity. It should be emphasised that hyperactivity is not merely high activity but refers to extreme activity levels in inappropriate contexts. While owners might only judge the amount of activity, trainers can assess its relevance to the situation.

We need to consider that there could be a difference between dogs that have a trainer evaluation and those that do not. For example, dogs with trainers might be taken to various places by their owners and may encounter more diverse situations than dogs that do not attend a dog school. This exposure to training/diverse situations could potentially lead to different behavioural responses compared to dogs that do not attend dog training programs. We observed comparable correlation coefficients regarding the ratings in the total sample and the subsample of owners, except for inattention ratings, where we found a somewhat higher correlation coefficient for the ratings of dogs with trainers. Owners who sought professional training for their dogs might possess a more nuanced understanding of attention due to their interaction with trainers. This exposure could provide them with insights into their pets’ behavioural patterns and tendencies, allowing for more precise evaluations. Engaging in group classes with their dogs, owners might also have greater experience of comparing their dogs’ attention to others’, which may result in more accurate evaluations. During training situations, dogs’ attention level is crucial and may be exhibited through different tasks, which enables owners to assess it more precisely. Interestingly, trainers’ assessments appear to cover a wider spectrum of inattention scores (Figure 4); for instance, trainers rated numerous dogs’ inattention higher than 10, while only one owner did so.

Importantly, the training level had only a small effect on the results of the tests, which met our expectations. Our aim was to develop a test battery that was largely independent of the dogs’ training level. Our results indicated that dogs with lower training levels left their positions more often. This is not surprising as the sit test can be regarded as a basic and common task that all owners teach their dogs, and it can be practised and enhanced with consistent training. With this test, we aimed to capture the human ADHD symptoms, i.e., “fidgets, bustles, or squirms in seat” and “often leaves seat” [1,2], and we assumed that this task would be especially challenging for dogs with extreme hyperactivity scores.

Our research is one of the first to examine test–retest reliability in the study of dog ADHD-like behaviour, the measurement of which is often neglected. As we expected, test–retest reliability demonstrated moderate consistency in the case of the looking at the experimenter variable in the attention test and strong to almost-perfect consistency across all other tests. These results indicate that our test battery is consistent in measuring inattention, hyperactivity, and impulsivity in dogs. This is in line with test–retest results for measuring dogs’ activity and impulsivity in the Activity–Impulsivity Behavioural Scale, which demonstrated excellent test–retest reliability [27]. We found lower consistency in measurements of attention, similarly to our previous questionnaire studies [19,25]. This lower consistency may be attributed to the inherent difficulty in evaluating attention, as also suggested by findings from human ADHD. These studies show that agreements for internalising behaviours such as inattention are generally lower than agreements for externalising behaviours such as hyperactivity and impulsivity, which are easier to observe and evaluate [53,54]. This explanation is further supported by the results for the agreement measurements of the ADHD questionnaire scores in the case of dogs, where owner–trainer agreement for the inattention scale was found to be lower than owner–trainer agreements for hyperactivity and impulsivity [19,25]. It should be noted that we applied a longer test–retest interval than usual, yet our tests still showed notable consistency, indicating that they can be used for longitudinal studies of inattention, hyperactivity, and impulsivity, offering insights into how these traits change over time.

In summary, we managed to develop a robust behavioural test battery that was as unaffected by environmental factors as possible, making it adaptable to a variety of settings, requiring no specialised facilities, and allowing for immediate evaluation with minimal equipment requirements.

## 5. Conclusions

We have successfully designed a comprehensive behavioural test battery which demonstrates significant correlations with both owner- and trainer-rated inattention, hyperactivity, and impulsivity scores in DAFRS [19]. This provides further validation for DAFRS and suggests that the tests of the test battery are able to effectively capture inattention, hyperactivity, and impulsivity in dogs. The current behavioural test battery can potentially contribute to the development of standard, objective diagnostic methods if the human-analogue of ADHDcan be diagnosed in dogs.

## Figures and Tables

**Table 1:** Behavioural variables measured in the tests, their definitions, and units of measurement. “s” indicates seconds.

Test	Variable	Definition	Unit
Leash test	Movement	Duration of moving and changing location (e.g., going around the owner) and moving without changing location (e.g., moving one leg, creeping, throbbing, changing position) except moving the head/ears and wagging the tail	s
Plush dog test (first and second part combined)	Movement	Duration of moving and changing location (e.g., going around the owner) and moving without changing location (e.g., moving one leg, creeping, throbbing, changing position) except moving the head/ears and wagging the tail	s
Sit test	Leaving position	Frequency of leaving the sit position; getting up or lying down. (It was not counted when the dog lay down after the first sit command.)	number
Attention test	Looking at experimenter	Duration of looking at experimenter and/or the manipulation of containers by experimenter	s

**Table 2:** Spearman’s correlations of the owner- and trainer-rated DAFRS ADHD subscale scores and behavioural test variables, accounting for training status. Sample sizes indicate the total sample of dogs with owner ratings (*n* = 59) and a subsample (*n* = 38) with both trainer and owner ratings. *p*-values are adjusted *p*-values based on Benjamini–Hochberg correction. *p*-values are indicated based on significance level as * *p* < 0.05, ** *p* < 0.01, and *** *p* < 0.001.

Spearman’s Correlations of ADHD Subscale Scores, Behavioural Test Variables, and Training Status					
Correlations	Sample Size	Owner Rating		Trainer Rating	
		*r*	*p*-Value	*r*	*p*-Value
Inattention score—Looking at experimenter in the attention test	*N* = 52	−0.365 *	0.009		
	*n* = 33	−0.429 *	0.013	−0.421 *	0.014
Hyperactivity score—Movement in the leash test	*N* = 59	0.383 **	0.004		
	*n* = 38	0.325 *	0.041	0.567 ***	<0.001
Impulsivity score—Movement in the plush dog test	*N* = 59	0.493 ***	<0.001		
	*n* = 38	0.481 **	0.003	0.519 **	0.001
Hyperactivity score—Leaving position in the sit test	*N* = 59	0.622 ***	<0.001		
	*n* = 38	0.626 ***	<0.001	0.590 ***	<0.001
Partial correlation by training; Hyperactivity score—Leaving position in the sit test	*N* = 59	0.582 ***	<0.001		
	*n* = 38	0.530 **	0.001	0.539 **	0.001

## Data Availability

The original data presented in the study are openly available in FigShare at https://doi.org/10.6084/m9.figshare.25931758 (accessed on 29 May 2024).

## Supplementary Materials

The supporting information regarding the attention test distractor sound can be downloaded at FigShare at https://doi.org/10.6084/m9.figshare.25931791 (accessed on 29 May 2024).
